# Metrology in sEMG and movement analysis: the need for training new figures in clinical rehabilitation

**DOI:** 10.3389/fresc.2024.1353374

**Published:** 2024-01-29

**Authors:** Roberto Merletti

**Affiliations:** LISiN, Department of Electronics and Telecommunications, Politecnico di Torino, Italy

**Keywords:** surface electromyography, sEMG, metrology, training, education, movement analysis, rehabilitation, physical therapy

## Abstract

A new educational curriculum for the next generation of physical and occupational therapists is urgent in order to manage the recent fast advances in sensors, measurement technologies and related instrumentation. This is required by the growing role of STEM in rehabilitation, kinesiology, and sport sciences. Surface EMG technology is used in this work as a representative example of similar problems present in movement analysis, exoskeletons, and many other fields. A review of the most relevant articles and international projects in the field of interfacing physical therapy with measurement technology for quantitative assessment of outcome is presented. It is concluded that a new generation of educators is needed as well as a Ph.D. and/or a clinical doctorate degree in physical therapy, still lacking in many countries. It is urgent to consider knowledge translation since it will take many years before any recommended change in teaching will be accepted and show some effect. A call for a “white paper” on rehabilitation metrology is highly auspicable.

## Introduction

Rehabilitation Engineering is usually associated to assistive technology. An often neglected area of great importance concerns the metrology of recovery or of disease progression to quantify the effectiveness of interventions in physical medicine and rehabilitation (PMR). This area is the foundation of Evidence Based Practice (EBP) in rehabilitation and has been addressed in a previous more general Frontiers project https://www.frontiersin.org/research-topics/23715/ and by the TEACH Erasmus Plus European project http://teacherasmusplus.eu/.

In the sub-field of motor rehabilitation, EBP in PMR is largely based on biomechanics, kinesiology, and surface EMG (sEMG) (1–4).

**Figure 1:**
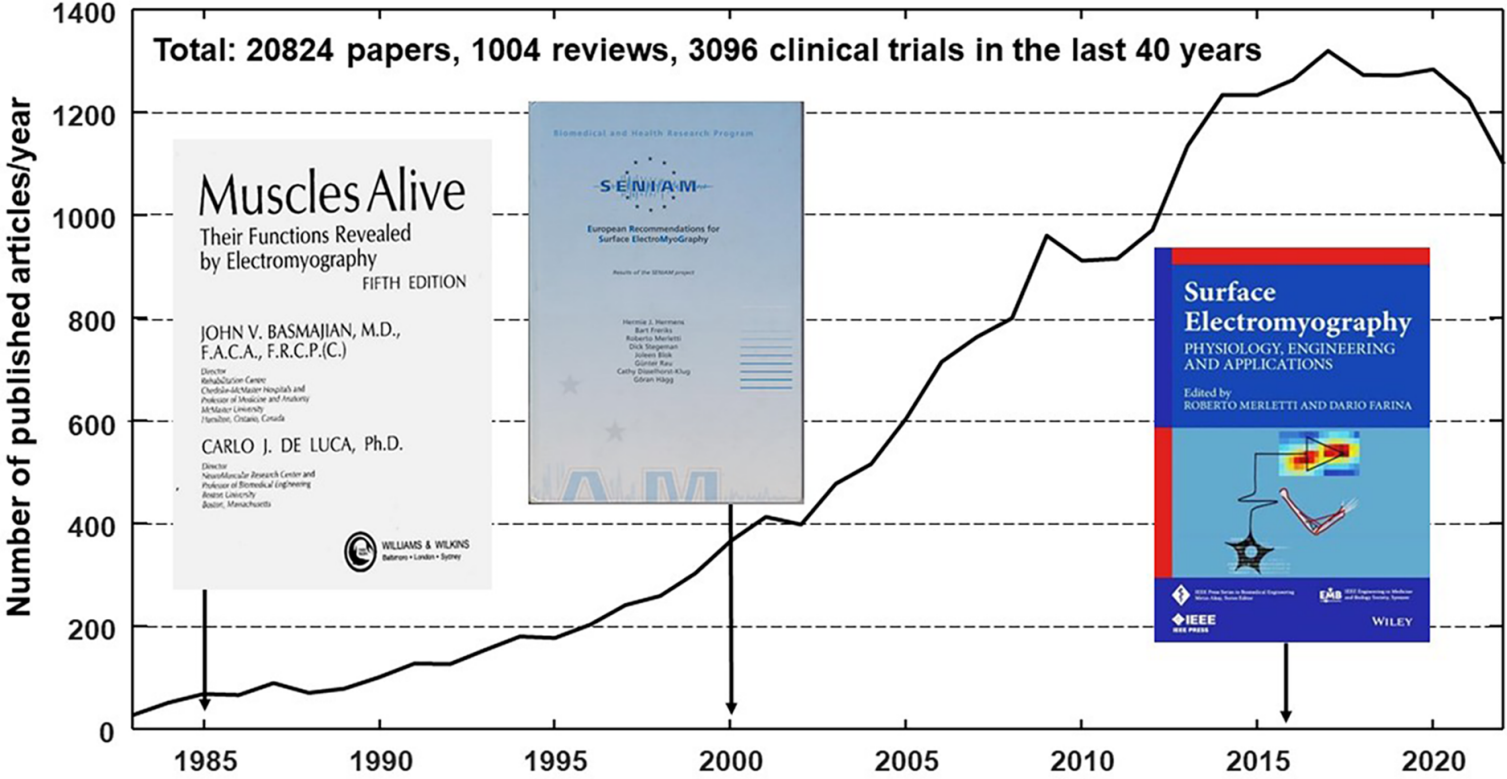
Rate of publications/year provided by a pubmed search using keywords (“surface electromyography” OR “surface EMG” OR sEMG OR HDsEMG) AND (rehabilitation OR sport OR ergonomics OR “movement analysis”) contained in title or abstract and limited to human subjects in the last 40 years (October 2023).

We focus here on sEMG measurements as a representative example. Similar considerations apply to other examples. Figure 1 shows three milestone textbooks and the large number of articles in the field; however, a similar search on EMBASE limited to clinically relevant journals resulted in only 21 clinical trials indicating that most clinical trials using sEMG were not submitted to (or accepted by) these journals.

It is urgent to consider knowledge translation since it will take many years before any recommended change in teaching will be accepted and will have some effect. In the meantime the educational gap will widen considerably.

Collecting evidence to plan treatment and monitor patient's performance implies making measurements. Making measurements implies familiarity with the physical quantities to be measured, with their measuring methods and the instruments used, their operational principles, their proper use and limitations/errors in the areas of biomechanics and electrophysiology, among others.

This implies competence, good training and some familiarity with science, technology, engineering and mathematics (STEM) necessary to collect evidence (2, 5–11). Evidence is also provided by scale evaluations and questionnaires; however quantification, repeatability and bias may be an issue in this case (3, 4).

Biomechanical measurements deal with quantification of movement and forces which we can see or feel. Electrophysiological measurements deal with signals that we can neither see nor perceive and whose features cannot be immediately visualized. They must be measured with suitable instruments and imply processing (12). In addition, both measurement types require time and multidisciplinary competences both of which are rarely available in the clinical world while they are more common in research institutions (4, 8, 9, 13, 14). This is one of the many reasons for the widening gap between the available sEMG knowledge/technology and its clinical application (2, 14, 15). Other reasons have been discussed elsewhere (16, 17).

The metrology of sEMG is a good example of EBP because (a) sEMG is one of the most information-rich bioelectric signals (like ECG and EEG), (b) this is a field where the gap between scientific knowledge and clinical practice is very wide, (c) extensive free educational material is available, (d) kinesiological sEMG is fundamental in gait and movement analysis and in exercise/sport physiology and, (e) potential developments are huge and unchartered and “*we are just scratching the surface*” (18). Other topics, such as exoskeletons, biomechanical analysis by IMU or stereofotogrammetry, proper applications of isokinetic machinery and force measuring braces, etc. are equally important.

The sEMG “signal”, like EEG, is actually an electrical image produced on the skin, evolving in time and sampled in time and space using electrodes whose number ranges from two (bipolar) to hundreds (High Density sEMG, HDsEMG). This image represents the algerbraic summation of the surface voltage distributions produced by the motor unit action potential trains of the active motor units located within a “detection volume” below the electrode array. It is affected by a variety of anatomical, physiological and geometrical factors (19). Unraveling these factors, and understanding which combination(s) of physiological factors might be responsible for a particular change of image or bipolar signal, is a difficult but irresistible challenge for engineers and also one of the reasons for the huge number of engineering papers which remain unaccessible to most clinical professionals [physiatrists, physical and occupational therapists (PTs and OTs), neurphysiology technicians, clinical technologists] despite recent efforts [www.robertomerletti.it/en/emg/material/teaching/, https://isek.org/isek-jek-tutorials/, https://isek.org/cede-project/].

Since the beginning, a century ago, EEG has been presented as mutichannel or as an image (2-D signal with a time-evolving 2-D power specrum) while sEMG has been presented mostly as a single bipolar channel (1-D signal) per muscle without exploiting or teaching its information-rich spatio-temporal nature until recently.

For decades PTs have been taught to use their “*healing hands*” and “*based the pride of their profession on ‘hands-on’ interventions*” (20). In the future clinical professionals will use instruments to measure physical quantities and will interact with robots (or cobots) since, “*…the patient-robot interaction is more effective than the patient-therapist interaction becase a therapist can never be as sensitive as the sensors of the robots and the timing and shaping of assistance by the therapists can never be as finely tuned as the assistance provided by the robot*” (20). This statement is now supported by the recent review of Huo et al. (21).

Artificial intelligence (AI) can merge information from movement, HDsEMG, EEG, and other sources to adapt models, build “digital twins”, use them to interpret and respond to the patient's behavior (22, 23) and make decisions. Competence to make measurements and familiarity with mathematical models, robots, and AI will be required from the clinical professionals who are entering their academic training today. They will be working with these tools. Sharing a common language with rehabilitation engineers is, and will be, an absolute requirement for PTs and OTs (11) implying some degree of STEM education allowing them to measure the effecs induced by therapy or disease progression, as required by insurance companies and National Health Services.

A large portion of the sEMG clinical literature reports small clinical studies mostly focused on testing a new device or a new signal processing technique on a few healthy subjects or, more rarely, on pathological subjects. Large studies and approved protocols are lacking and they cannot be proposed and carried out only by engineers or research oriented operators. There is a need for new figures who have the time and the interdisciplinary competence required to carry out these studies.

## The need for education of new professional figures

In the surveys carried out within the framework of the European Erasmus Plus “TEACH” project (http://teacherasmusplus.eu/) involving 104 undergraduate teachers in health sciences from twelve different countries in Europe, only 17% received some education about instrumented analysis techniques (1). The survey published by Estomba et al. (24) indicated that out of 112 rehabilitation resident doctors, only 18.8% declared having sufficient training resources available. Likewise, the results of a questionnaire administered in the context of the EU Leonardo Da Vinci Program “Biomechanics4rehab” indicated that, of 184 Rehabilitation specialists from across Europe (contacted by the European Society of Physical Medicine and Rehabilitation: ESPRM), 87% had not completed any training in Biomechanics and/or analysis methodologies in the last 10 years, and that 56% would not know how to interpret the results of a biomechanical analysis (1).

In his 2016 and 2017 Editorials published in Physical Therapy, A. Jette outlined that “*There are major gaps in our scientific knowledge; however, even more disturbing is the fact that an enormous amount of existing scientific knowledge remains unused in practice*.” and that “ … *the frequently quoted statement about the lag time between publication and adoption of research—only 14% of original research is applied for the benefit of patient care, and that takes 17 years—is alarming enough*.”, and that “*In the 21st century, the field of rehabilitation will certainly need to continue to focus on developing new clinical innovations based on scientific evidence (combating ignorance) but will also need to develop new and creative initiatives to overcome ineptitude by disseminating rehabilitation innovations at a much faster pace than we have in the past*.” (8, 9). This will be the responsibility of educational institutions and implies to have competent teachers with time to study and do research. But, even so, time to set up instrumentation, perform measurements, process and interpret results may be unavailable because of the busy schedule of today's PTs and OTs (3). Then, how can changes be implemented? The proposed way is through teaching and training a new generation of PTs, OTs, and clinical research professionals. This implies a new generation of faculty members, as concluded by Helgøy et al. who recently indicated that “…*research-based education should be increased among both faculty members and students*” (25). Similar conclusions had been reached by Pearce et al. (26) who analyzed “Train-the-Trainers” courses for MDs and health professionals. Many other authors addressed the issue of perceived barriers to EBP or sEMG use (27–31).

Higher level training, including STEM concepts, is difficult to implement when (as in many countries) a PhD or clinical doctorate in physiotherapy or rehabilitation sciences is not available and academic careers in these fields are not accessible. The educational process is very heterogeneous in different countries. The need to harmonize educational standards across countries has been identified by the European Union of Medical Specialists (UEMS) and the International Society of Physical Rehabilitation and Medicine (ISPRM) that defined standards but, given the growth rate of new technologies and assessment methods, they must be updated frequently (1).

**Figure 2:**
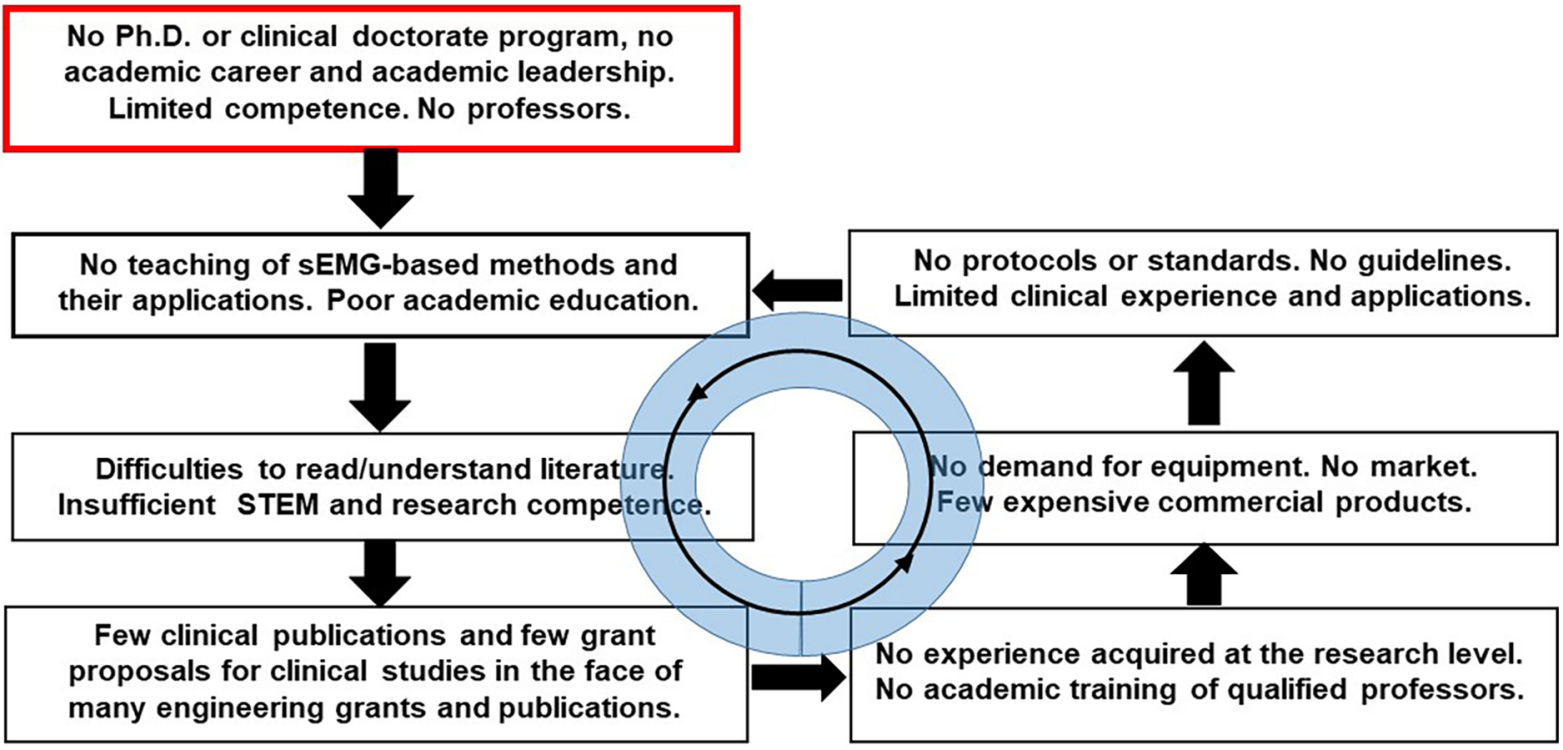
The vicious circle resulting, in many EU countries, from the lack of a Ph.D. degree in physiotherapy.

The situation is somewhat different in the USA were undergraduate studies typically take four years and physical therapy graduate school takes three more years. In EU countries undergraduate education takes three years, an MS takes two additional years while a Ph.D. or a clinical doctorate is available only in a few countries. This situation has been discussed in the Frontiers open book [https://www.frontiersin.org/research-topics/11157/] (19 articles, 74 authors, 150,000 views, 25,000 downloads) and creates the vicious circle described in Figure 2. Traditional and free online educational resources have been available for many years as books [see Figure 1 and (32)], tutorials and webinars published by the International Society for Electrophysiology and Kinesiology (ISEK) [https://isek.org/isek-teaching-repository/, https://isek.org/isek-jek-tutorials/, https://isek.org/cede-project/, https://isek.org/isek-journal/], peer reviewed articles (15, 33), presentations [https://guides.library.unisa.edu.au/SystematicReviews/Home], and free teaching modules [www.robertomerletti.it/en/emg/material/teaching/].

Examples of teaching signal processing concepts have been provided in the supplementary material of (2) and in technical notes [https://www.robertomerletti.it/en/emg/material/tech/].

These are positive initiatives, but will they change the deeply rooted attitudes described by A. Jette as “*Publishing our work in journals is essential but publication of research is not, by itself, sufficient if our goal is to change clinical practice. People follow the lead of other people they know and trust when they decide whether to take up an innovation and change the way they practice*.” (8)? Probably not, unless a teaching revolution is implemented, including structured online education as part of a study curriculum.

As indicated by S. Peacock “*It is hoped that physiotherapists will benefit from the use of online learning, not only within formal types of professional education but also from the opportunities online communities provide to access, debate and share knowledge and examples of good practice*.” (28). However, ten years later S. Barradell et al. still pointed out that “*Contemporary and future physiotherapists are, and will be, presented with challenges different to their forebears. Yet, physiotherapy tends to remain tied to historical ways of seeing the world: these are passed down to generations of physiotherapy graduates*.” (27).

The sEMG science (like the EEG science) can be quite intimidating and overwhelming for those lacking some STEM background. Digital education (such as some online courses or ISEK tutorials) maybe less stressful. Ødegaard and co-workers interviewed 12 teachers in physiotherapy education and concluded that “…*physiotherapy teachers are skeptical about digital education, primarily viewing it as a threat to established teaching practices*.” and “*….the findings demonstrate a potential for digital transformation in physiotherapy education, which can be released by informing the current teaching practices with evidence from research showing how the use of digital technology can improve teaching and learning in physiotherapy education.*” (34). Furthermore, as early as 1997, Brandt and Pope pointed out that “*No topic is likely the focus of more discussion but less productive action than technology transfer. The reason is simple: technology transfer is difficult and problematic.*” (35). In addition, as hinted by A. Jette, 20 years later, problems may also be due to “*Local worlds*” that “*rule the clinical networks in which we live and practice and have substantial influence on the clinical behavior of rehabilitation professionals*.” (8).

These difficulties and problems have not yet been seriously addressed in the sEMG field. Although there are technical tutorials and consensus papers prepared by the Intern. Society of Electromyography and Kinesiology (https://isek.org/isek-jek-tutorials/, https://isek.org/cede-project/) to explain and guide the correct detection and interpretation of sEMG and HDsEMG, clinical practical guidelines, prepared by expert clinicians, are lacking.

Correcting this situation implies a joint effort of engineers and PTs/OTs. Filling this gap requires time, experience and expertise that the traditional PTs do not have and cannot acquire in a 3-year BS program. Higher levels of education are needed. A relevant point to discuss is whether a basic knowledge in STEM should be a requirement for entering a BS program or should be provided within it by properly trained teachers.

Although these problems are particularly serious in rehabilitation and sport physiotherapy and in movement sciences, they are not exclusive of these fields. The Frontiers Project https://www.frontiersin.org/research-topics/16682/ (15 papers, 47 authors, 145,000 views) addressed them in the area of pseudo-neuroscience and neuromyths. In particular, Carboni et al. pointed out that “*Neuroscience research has produced a great deal of information that can potentially help in the transformation of education, promoting interventions that help in several domains including literacy and math learning, social skills and science*.” (36).

Why and how much should a rehabilitation clinical professional know about measuring muscle activity timing and coordination, muscle synergies, sEMG spectrum and EEG-EMG coherence, sEMG image features, sEMG decomposition, location of the innervation zone of a muscle, or muscle fiber conduction velocity? Certainly not as much as rehabilitation engineers but enough to interact with them and use instruments and software with competence and confidence in clinical environments. Therefore, teaching should be tuned to meet the needed initial STEM background and the STEM-based new technologies.

The need for competence in STEM and the ability to perform measurement and analysis of results, is increasing in most specialties of Health Sciences and its acquisition does not fit in the general European 3-years BS curriculum. Alternatives are (a) acquiring this competence in higher degree curricula (MS, PhD) or, (b) considering specific new clinical professional figures such as the clinical technologists mentioned in (37) and in section 4.3 of (2). Unfortunately, a PhD degree in physiotherapy is available only in a few countries and new technical figures are often not seen favorably. As a consequence, a new generation of professors with multidisciplinary competence is not being formed in academic institutions and the cultural gap described above increases rapidly, preventing patients from taking full advantage of recent technologies for diagnosing, monitoring, and proper treating of their condition.

## Limitations and conclusions

This article summarizes the most relevant literature concerning the need for better academic training of clinical professionals and movement scientists in the proper use of technology, taking sEMG metrology as an example. Discussion about this and other metrologies will hopefully come from other contributors. Other debate arenas and forms of “teaching” are present on YouTube and are not discussed in this work because are not subjected to peer review and not always appropriate or correct.

Many more articles and examples are available and the perception of the problem is much more extensive than reported here. The barriers to its correction are many and of different nature. Their removal requires strong action from motivated stakeholders. A “white paper” on metrology in rehabilitation is highly auspicable.

While a BS or MS degree is likely sufficient to certify clinical competence in the “traditional” PT/OT professions, new clinical figures with a much greater capability to manage new methods and instruments together with rehabilitation engineers, are required and must be trained by suitably motivated professors. While it is true that “…*consensus on how to address these problems, how to form teachers, how to disseminate knowledge and improve academic education, … is lacking*” (2), it is also true that incorporating advances in technology is placing heavy burden and responsibilities on health sciences faculty to teach material beyond their original expertise. New interdisciplinary teaching resources are needed. As indicated by Trumbower and Wolf, “*Should our profession provide interdisciplinary resources and skills necessary for physical therapists to meet these types of new responsibilities? Yes. Provocative discoveries and challenges come with new opportunities, and physical therapists must be prepared to act on them*.” (10). These authors also provide examples of US Universities that have positively addressed the problem. Their experience and the experience acquired in different countries (offering or not a Ph.D. degree) should be analyzed.

## Scope statement

An often neglected area in Rehabilitation Engineering is metrology of recovery and of disease progression to quantify the effectiveness of interventions. This area is the foundation of Evidence Based Practice (EBP) in rehabilitation and concerns measurement technologies and the related instrumentation. The fast development of technology requires new competences by the next generation of physical and occupational therapists in the fields of science, technology, engineering and mathematics (STEM), which, in turn, requires a new educational curriculum and a new generation of educators as well as a Ph.D. and/or a clinical doctorate degree in physical therapy, still absent in many countries.

The metrology of surface electromyography (sEMG) is discussed as a good example of EBP because (a) sEMG is one of the most information-rich bioelectric signals (like ECG and EEG), (b) the gap between scientific knowledge and clinical practice in this field is very wide, (c) extensive free material is available and (d) potential developments are huge and unchartered. Why and how much should a rehabilitation clinical professional know about measuring muscle timing and coordination, muscle synergies, spectral analysis of sEMG, EEG-EMG coherence, etc. A review of the main literature about the need for addressing this educational problem is provided.
